# The developmental–diagnostic gap: cultural invisibility and delayed recognition of prenatal alcohol exposure

**DOI:** 10.3389/fpsyg.2026.1836200

**Published:** 2026-05-13

**Authors:** Maria Kamecka

**Affiliations:** Independent Researcher, Doncaster, United Kingdom

**Keywords:** developmental–diagnostic gap, diagnostic delay, early identification, FASD, neurodevelopment, neurodevelopmental disorders, placenta–brain axis, prenatal alcohol exposure

## Abstract

Foetal alcohol spectrum disorders (FASD) originate during prenatal development, with recognition typically occurring only after behavioural differences become apparent. This delay reflects a temporal disconnect between early neurodevelopmental disruption and later clinical identification, highlighting a structural limitation in diagnostic approaches that rely primarily on observable outcomes. This paper introduces the concept of a developmental–diagnostic gap as a framework for understanding this disconnect. Drawing on existing literature, it integrates neurobiological, developmental, and sociocultural perspectives to examine how prenatal alcohol exposure affects brain development prior to behavioural recognition. Neurobiological mechanisms, including disruptions to neuronal migration and synaptic development, are considered alongside emerging molecular and epigenetic evidence. The placenta–brain axis is also explored as a potential pathway linking the maternal environment to foetal neurodevelopment. The framework highlights how reliance on behavioural presentation may contribute to delayed recognition, misinterpretation, and the accumulation of secondary developmental consequences. Cultural factors, including the normalisation of alcohol use and stigma surrounding maternal behaviour, further limit the visibility of early risk and contribute to under-identification. The developmental–diagnostic gap is presented as a conceptually grounded, empirically testable framework. It supports investigation of early biological indicators, including placental signalling and prenatal biomarkers, through longitudinal and interdisciplinary research designs. Addressing this gap has important implications for research, clinical practice, and public health. Earlier recognition of neurodevelopmental disruption may improve developmental outcomes by enabling timely, developmentally informed support and reducing the accumulation of secondary harm.

## Introduction

Neurodevelopmental differences originate early in development but are typically recognised only after they are expressed behaviourally. In the context of prenatal alcohol exposure, this temporal gap between biological origin and clinical recognition presents a significant challenge. Foetal alcohol spectrum disorders (FASD) are associated with a range of cognitive, behavioural, and regulatory difficulties, and they remain widely under-recognised despite affecting a substantial proportion of the global population (Popova et al., 2017).

Existing research has largely focused on the effects of alcohol on foetal development and the behavioural characteristics observed later in life. However, less attention has been given to the developmental period in which these differences are present but not yet behaviourally expressed. As a result, many individuals are identified only after difficulties emerge, often within frameworks that prioritise observable behaviour over underlying neurodevelopmental processes.

This paper introduces the concept of a “developmental–diagnostic gap” as a framework for understanding the temporal disconnect between early neurodevelopmental disruption and later behavioural recognition in FASD. Neurobiological effects, behavioural outcomes, and diagnostic challenges have been widely studied, but they are often addressed separately. Bringing these strands together, the present work proposes a conceptual model linking early biological change, delayed clinical recognition, and sociocultural factors contributing to under-identification.

This perspective highlights that the cultural invisibility of prenatal alcohol exposure contributes to delayed recognition and the misinterpretation of neurodevelopmental differences, with important implications for early intervention and long-term outcomes.

Adopting a conceptual approach, the paper draws on selected literature to develop an integrative theoretical framework.

Prenatal alcohol exposure is known to disrupt foetal brain development through a range of neurobiological mechanisms, including alterations in neuronal migration, synaptic development, and executive functioning pathways (Guerri et al., 2009; Riley et al., 2011).

## Neurobiological effects of prenatal alcohol exposure

Prenatal alcohol exposure occurs during a period of rapid and highly sensitive brain development, when foundational neural structures and connections are being formed. Alcohol readily crosses the placenta and reaches the developing foetus, where it can interfere with critical neurodevelopmental processes. Unlike the mature brain, the foetal brain lacks the capacity to compensate for such disruptions, making it especially vulnerable even to low levels of exposure.

At the cellular level, alcohol affects several key processes essential for typical brain development. It can impair neuronal proliferation, resulting in a reduced number of neurons, and disrupt neuronal migration, leading to the misplacement of developing cells within the brain. In addition, alcohol exposure has been associated with increased apoptosis, or programmed cell death, further reducing neural cell populations. These effects are compounded by disruptions in synaptogenesis, the process through which neurons form connections. Alcohol exposure is also associated with alterations in key neurotransmitter systems, including gamma-aminobutyric acid (GABA) and glutamate, both of which are critical for neural communication and network stability (Guerri et al., 2009; Mattson et al., 2019; Bariselli and Lovinger, 2021; Wilhelm and Guizzetti, 2016; Center on the Developing Child, 2007). Prenatal alcohol exposure has also been linked to molecular and epigenetic alterations, including oxidative stress pathways, changes in gene expression, and early epigenetic reprogramming, which may further contribute to disrupted neurodevelopmental processes (Sessa et al., 2025).

These disruptions are not confined to a single region but affect multiple areas of the brain. Structural and functional alterations have been observed in the prefrontal cortex, which supports executive functioning and decision-making. The hippocampus, central to learning and memory, is also affected, along with the cerebellum, which contributes to coordination and cognitive processing. Changes in the corpus callosum may disrupt communication between the brain’s hemispheres (Riley et al., 2011). As a result, prenatal alcohol exposure leads to widespread and persistent changes in brain organisation and function.

Crucially, these alterations occur during the earliest stages of development, when the brain’s basic architecture is being established. Even small disruptions can have cascading effects over time, influencing how neural systems develop, interact, and respond to environmental demands. These changes may not be immediately visible at birth, but they form the foundation for later cognitive, behavioural, and regulatory differences that emerge as developmental demands increase.

These early alterations shape neural organisation before becoming behaviourally visible. In doing so, they establish the biological foundation for later neurodevelopmental differences, leaving an extended period during which these differences remain largely unrecognised. This period of biological change in the absence of behavioural identification represents the early phase of the developmental–diagnostic gap, in which underlying neurodevelopmental disruption is present but not yet captured within clinical or behavioural frameworks. As a result, the origins of neurodevelopmental difference are temporally separated from their later recognition, contributing to the diagnostic delays explored in this paper.

## The invisible phase of neurodevelopment

Building on these early neurobiological changes, the behavioural characteristics later associated with foetal alcohol spectrum disorders reflect underlying differences that emerge during early brain development (Guerri et al., 2009; Riley et al., 2011). These differences are present from the earliest stages of neurogenesis, prior to behavioural identification. During this period, the brain develops alongside other physiological systems through processes that are both complex and highly sensitive to environmental influences.

From a clinical perspective, however, this early phase of neurodevelopment often remains unrecognised. In the absence of distinctive physical features, prenatal alcohol exposure may not be considered, and neurodevelopmental differences may go unidentified. As a result, these early changes persist without clinical acknowledgement, despite already shaping neural organisation and function.

Diagnosis of FASD frequently occurs later in development, particularly when behavioural differences become more apparent (Popova et al., 2017; Subramoney et al., 2018). By this stage, difficulties in areas such as executive functioning, emotional regulation, and adaptive behaviour begin to emerge. These are often interpreted within behavioural or psychological frameworks, rather than being understood as the outcomes of earlier neurodevelopmental processes (Mattson et al., 2019). In this way, the absence of visible physical markers can contribute to the assumption that no underlying condition is present, delaying recognition of the neurobiological origins of these differences.

These observations highlight a temporal disconnect between early neurodevelopmental disruption and its later recognition within clinical frameworks. This disconnect forms a central component of the conceptual framework proposed in this paper.

## The developmental–diagnostic gap

The concept of a developmental–diagnostic gap refers to the temporal disconnect between early neuro-developmental disruption and its later recognition within clinical and behavioural frameworks. In the context of foetal alcohol spectrum disorders, neurobiological changes occur during the earliest stages of brain development, yet identification typically relies on the emergence of observable behavioural differences.

This reliance on behavioural presentation creates a delay between the presence of underlying neurodevelopmental alterations and their recognition in clinical practice, particularly in the absence of distinctive physical features. During this early developmental period, physical markers of foetal alcohol exposure may be minimal or absent, despite ongoing disruption to brain development. As a result, individuals may experience extended periods during which their difficulties remain unexplained, misunderstood, or attributed to alternative conditions. This delay can contribute to misdiagnosis, including classification under other neurodevelopmental or behavioural disorders, and may obscure the neurobiological origins of these differences.

The developmental–diagnostic gap therefore reflects a structural limitation within current diagnostic approaches, which prioritise behavioural expression over early neurodevelopmental indicators. Given the prevalence and potential severity of foetal alcohol spectrum disorders, this gap highlights the need for a conceptual shift towards earlier recognition of neurodevelopmental disruption and a more integrative understanding of its biological, behavioural, and sociocultural dimensions.

The significance of this gap lies in its timing. It spans a critical period of brain development, during which early neurobiological disruption continues to shape neural organisation and functional outcomes. Foetal alcohol spectrum disorders emerge during prenatal development but are typically identified only once behavioural differences become apparent. Delayed recognition therefore reflects a lag in detection.

**Figure 1:**
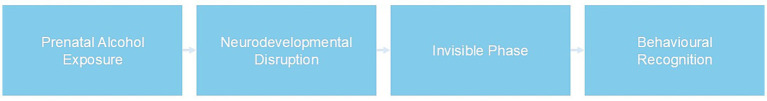
Developmental–diagnostic gap in prenatal alcohol exposure.

This distinction carries important implications for developmental outcomes. In the absence of early identification, neurodevelopmental differences continue to interact with environmental demands over time, potentially contributing to cumulative difficulties across cognitive, behavioural, and adaptive domains. As a result, early neurodevelopmental disruption remains unaddressed during a formative period in which intervention may be most effective. To clarify this temporal progression, Figure 1 illustrates the proposed developmental–diagnostic gap, highlighting the progression from early neurodevelopmental disruption to later behavioural recognition.

This figure illustrates the temporal disconnect between early neurodevelopmental disruption and later behavioural recognition, highlighting the prolonged “invisible phase” during which underlying impairments remain unrecognised.

Understanding this gap highlights the need to examine the biological mechanisms through which early neurodevelopmental disruption is mediated. One such pathway may involve the placenta, which plays a central role in regulating the intrauterine environment.

The developmental–diagnostic gap extends beyond existing concepts such as diagnostic delay by explicitly linking early neurodevelopmental disruption to later clinical recognition within a single temporal framework. While diagnostic delay describes a lag in identification, it does not account for the biological processes occurring prior to behavioural manifestation. The present framework integrates early neurobiological change, delayed recognition, and subsequent developmental outcomes, offering a more comprehensive account of how neurodevelopmental differences emerge and are interpreted over time.

## The placenta–brain axis

**Figure 2:**
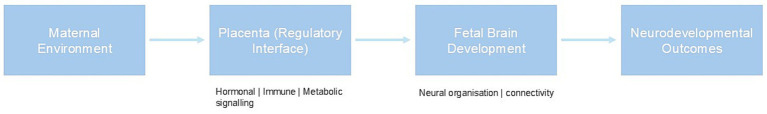
Placenta–brain axis in early neurodevelopment.

The placenta is increasingly recognised as an active contributor to foetal brain development, extending beyond its traditional role as a support organ (Rosenfeld, 2021). As the biological interface between mother and foetus, it regulates the intrauterine environment through endocrine, immune, metabolic, and vascular signalling. Through these processes, the placenta reflects key aspects of the environment in which the foetal brain is developing. Emerging research on the placenta–brain axis suggests that maternal experiences and exposures may be translated through placental processes into developmental signals that influence foetal brain development (Rosenfeld, 2021). As shown in Figure 2, the placenta functions as a regulatory interface between the maternal environment and foetal brain development, mediating hormonal, immune, and metabolic signalling processes.

This figure presents the role of the placenta as a regulatory interface between maternal environment and foetal brain development, mediating hormonal, immune, and metabolic signalling processes that shape neurodevelopmental outcomes.

This positions the placenta as a central component in neurodevelopmental research. Maternal stress, inflammation, and substance exposure may influence placental function, altering hormonal regulation, inflammatory signalling, blood flow, and nutrient delivery at critical stages of development (Bale, 2015). These changes do not determine outcomes in a simple or linear way but may contribute to vulnerability in neural systems linked to later cognition, emotional regulation, and behaviour. Increasingly, prenatal stress literature places the placenta at the centre of this developmental pathway, identifying it as one of the earliest biological routes through which environmental conditions may shape later psychiatric and neurodevelopmental risk (Bale, 2015).

In the context of prenatal alcohol exposure, the placenta may represent a particularly important and underexplored link between early exposure and later neurodevelopmental outcomes. Evidence suggests that alcohol can alter placental function and placenta–fetal brain signalling, raising the possibility that some effects of prenatal alcohol exposure may be mediated or modulated through placental mechanisms (Rosenfeld, 2021). These effects may involve molecular and epigenetic pathways, including oxidative stress and altered gene regulation, which have been identified as potential mechanisms linking prenatal exposure to later neurodevelopmental outcomes (Sessa et al., 2025). Placental measures are not currently used for diagnostic purposes but may offer a future direction for identifying early biological signals prior to the emergence of behavioural differences. The placenta therefore offers one of the earliest measurable points within the developmental–diagnostic gap, linking prenatal exposure to the later recognition of neurodevelopmental differences.

## Delayed recognition and misinterpretation of neurodevelopmental differences

Behavioural characteristics associated with foetal alcohol spectrum disorders are frequently interpreted through behavioural or psychiatric frameworks rather than being understood within a neurodevelopmental context (American Psychiatric Association, 2013). Difficulties with impulse control, emotional regulation, attention, and social understanding may present as “challenging behaviour,” often bringing individuals into contact with educational, clinical, or disciplinary systems that prioritise observable behaviour over its underlying neurobiological basis (Mattson et al., 2019; Riley et al., 2011).

Although these differences originate during early brain development, recognition is often delayed, particularly in the absence of visible physical characteristics. As a result, many individuals with prenatal alcohol exposure remain undiagnosed or receive alternative diagnoses, such as attention-deficit/hyperactivity disorder (ADHD), oppositional defiant disorder, or mood-related conditions, which may only partially reflect their neurocognitive profile (Mattson et al., 2019). This overlap in behavioural presentation contributes to diagnostic ambiguity and may obscure the underlying cause of the difficulties.

Current diagnostic frameworks for foetal alcohol spectrum disorders rely on a combination of clinical indicators, including growth deficiencies, craniofacial features, central nervous system impairment, and confirmed or suspected prenatal alcohol exposure (Sessa et al., 2022). However, these criteria rely heavily on observable characteristics, which may not be present in all individuals, particularly in cases without distinct facial features. This contributes to delayed or missed identification of underlying neurodevelopmental differences.

Within the context of the developmental–diagnostic gap, this delay in recognition reflects a mismatch between early neurodevelopmental disruption and later behavioural interpretation. Delayed recognition can have significant developmental consequences. Without an understanding of the neurobiological basis of these behaviours, responses are often framed in terms of discipline, motivation, or personality. Over time, individuals may internalise these interpretations, viewing their difficulties as personal failure rather than as expressions of underlying neurodevelopmental difference. This process has been linked to a range of secondary outcomes, including disrupted education, mental health difficulties, substance use, and increased vulnerability within the criminal justice system (Streissguth et al., 2004).

These outcomes are shaped not only by early neurodevelopmental differences but also by how those differences are recognised and responded to over time. When underlying neurodevelopmental processes are not identified, behavioural expressions may be interpreted in ways that do not reflect their origin. This can result in repeated mismatches between individual needs and environmental responses, contributing to patterns of exclusion, frustration, and negative self-perception. The long-term impact of prenatal alcohol exposure therefore reflects early biological disruption as well as the timing and nature of recognition.

## Secondary harm and developmental consequences

When neurodevelopmental differences remain unrecognised or are misinterpreted, individuals may experience secondary difficulties that extend beyond the original condition. These outcomes are not entirely attributable to prenatal alcohol exposure but often emerge through repeated interactions with environments that do not recognise or adequately respond to underlying neurodevelopmental needs (Streissguth et al., 2004). They may also reflect the cumulative effects of delayed diagnosis and unmet neurodevelopmental needs within educational, clinical, and social systems.

Difficulties with attention, impulse control, and emotional regulation may contribute to challenges within educational settings, where behaviour is frequently interpreted in disciplinary terms. This can increase the risk of school exclusion, disengagement from learning, and reduced educational attainment. Over time, repeated negative experiences may also shape self-perception, with individuals internalising a sense of failure or inadequacy.

Mental health difficulties are also more commonly reported, including anxiety, depression, and substance use. In some cases, behavioural challenges and unmet needs may lead to contact with the criminal justice system. Here, actions are often interpreted through social or moral frameworks rather than as expressions of neurodevelopmental difference (Streissguth et al., 2004). These pathways illustrate how misinterpretation can influence developmental outcomes in ways that extend beyond the initial neurobiological disruption.

Foetal alcohol spectrum disorders are irreversible; however, developmental trajectories remain flexible. Early recognition and appropriate support may significantly alter outcomes by reducing the accumulation of secondary harm. The brain remains plastic, especially in early life, and targeted interventions can support the development of adaptive skills, emotional regulation, and coping strategies. Early identification does not change the origin of the condition, but it can meaningfully influence its developmental course (Mattson et al., 2019).

## Cultural invisibility of alcohol-related harm

Despite extensive research on the effects of prenatal alcohol exposure, awareness of foetal alcohol spectrum disorders remains limited in both public and clinical contexts (Popova et al., 2017). Alcohol consumption is widely normalised across many cultures and embedded within long-standing social practices. While some communities abstain for religious or cultural reasons, in many settings alcohol remains a routine and socially accepted part of daily life. Within this context, the potential neurodevelopmental impact of prenatal exposure is often under-recognised or insufficiently understood.

This limited awareness is further influenced by stigma and sensitivity surrounding maternal behaviour during pregnancy. Discussions about alcohol use may be perceived as judgmental, which can discourage open disclosure in healthcare settings. As a result, reported alcohol consumption during pregnancy may not always reflect actual behaviour, particularly where individuals have been reassured that small amounts are unlikely to cause harm, reflecting inconsistencies in public health guidance (O’Leary et al., 2007). Cultural narratives, such as the belief that occasional drinking is acceptable, may further contribute to uncertainty and underreporting.

Normalisation, stigma, and uncertainty create conditions in which prenatal alcohol exposure may remain largely invisible. Efforts to avoid judgement are important for maintaining trust and engagement with healthcare services, but they may also limit opportunities for early identification and support. Within the context of the developmental–diagnostic gap, these cultural dynamics further contribute to the delayed recognition of neurodevelopmental differences by limiting the visibility of early risk factors. Consequently, the developmental impact of prenatal alcohol exposure may remain under-recognised, not due to a lack of evidence, but because cultural conditions limit its visibility. The absence of consistent and unified public health messaging regarding alcohol use during pregnancy represents a critical limitation in prevention, particularly given that foetal alcohol spectrum disorders are entirely preventable.

## Early identification as a neurodevelopmental opportunity

Framing foetal alcohol spectrum disorders within a neurodevelopmental context shifts the focus from late-stage behavioural management to earlier recognition of underlying brain differences (Mattson et al., 2019). This perspective highlights the potential for earlier intervention, particularly given that developmental outcomes remain modifiable despite the early and persistent nature of the condition. Early identification plays a critical role in shaping developmental trajectories by enabling timely and appropriate support.

Advances in neuroscience suggest emerging opportunities to improve early identification. Developments in neuroimaging, alongside research on placental signalling and prenatal biomarkers, suggest new possibilities for early identification. These advances may allow the detection of biological indicators of neurodevelopmental risk before behavioural differences become fully expressed (Rosenfeld, 2021). While these approaches are not yet sufficient for clinical diagnosis, they represent important directions for future research.

Progress in this area may benefit from integrating biological, clinical, and social perspectives to reduce the gap between early neurodevelopmental disruption and later recognition.

The importance of early identification lies in the brain’s capacity for adaptation. Neuroplasticity, particularly in early life, allows neural systems to reorganise in response to experience and intervention (Kolb and Gibb, 2011). When support is introduced early, it may strengthen adaptive pathways, support emotional regulation, and reduce the likelihood of secondary harm. Early identification cannot alter the origin of the condition, but it can meaningfully influence its developmental course.

Diagnosis becomes an opportunity rather than a label, enabling earlier understanding, more appropriate intervention, and improved developmental outcomes.

## Reframing behaviour through a neurodevelopmental lens

Current approaches to foetal alcohol spectrum disorders and related conditions are largely based on behavioural observation. While this supports identification, it often reflects the later expression of underlying processes, without capturing their developmental origins.

A neurodevelopmental perspective offers an alternative by focusing on how early brain development shapes cognition, emotional regulation, and interaction with the environment. This shift reframes interpretation, moving from surface-level behaviours to the underlying developmental processes that give rise to them.

Advances in neuroimaging and research on early biological pathways, including the placenta–brain axis, support the possibility of identifying neurodevelopmental differences earlier in life. These developments point towards a more integrated approach that combines behavioural observation with biological insight.

Within the context of the developmental–diagnostic gap, this perspective reduces reliance on later behavioural presentation and instead emphasizes earlier identification of underlying neurodevelopmental differences. It also raises broader questions about whether similar patterns of delayed recognition may exist across other neurodevelopmental conditions.

This shift positions behaviour as a later expression of earlier developmental processes, highlighting the importance of recognising neurodevelopmental differences at earlier stages.

## Discussion and implications

The concept of a developmental–diagnostic gap provides a framework for understanding delayed recognition in neurodevelopmental conditions. Its value lies in highlighting the importance of timing in how differences are identified and understood.

From a research perspective, the developmental–diagnostic gap directs attention towards the investigation of early biological indicators of neurodevelopmental difference. Pathways such as placental signalling, molecular mechanisms, and prenatal biomarkers represent promising areas for advancing earlier identification, particularly before behavioural patterns become established.

This framework can be examined through longitudinal and interdisciplinary research designs linking early biological indicators, including placental function and prenatal biomarkers, with later behavioural outcomes to assess the temporal disconnect proposed in this model. This may include examining discrepancies between early biological indicators and the timing of clinical diagnosis or identifying patterns in which neurodevelopmental disruption is present but not recognised within existing diagnostic frameworks. In addition, retrospective analyses of diagnostic pathways may help to identify patterns of delayed recognition and misinterpretation across developmental stages. These approaches enable empirical evaluation and refinement of the framework.

In clinical and educational contexts, this perspective emphasises the importance of interpreting behaviour within a developmental framework. This may support more responsive interventions and reduce reliance on approaches that focus solely on observable behaviour without considering underlying processes.

At a broader level, the developmental–diagnostic gap may extend beyond prenatal alcohol exposure, offering a lens through which delayed recognition can be examined across a range of neurodevelopmental conditions. Future research can apply this framework to examine how early biological differences interact with environmental and systemic factors across development.

The developmental–diagnostic gap provides a foundation for developmentally informed recognition of neurodevelopmental difference. It emphasises the need for earlier identification, integrative understanding, and context-sensitive responses across research, practice, and policy.

## Limitations

This paper adopts a conceptual approach and does not present original empirical data. While the developmental–diagnostic gap provides a framework for understanding delayed recognition, its application requires further empirical validation. In addition, the framework may not fully capture variability in diagnostic pathways, which are shaped by clinical, social, and systemic factors. The identification of early biological indicators remains an emerging area of research, and current methods are not yet sufficient for routine clinical application. Future research is needed to examine how this model can be operationalised and applied across different populations and contexts.

## Conclusion

The temporal disconnect between early neurodevelopmental disruption and later clinical recognition highlights a structural limitation in current diagnostic approaches, which prioritise observable outcomes over underlying developmental processes. The developmental–diagnostic gap offers a framework for understanding this delay by linking early neurobiological change to later behavioural identification.

By integrating neurobiological, developmental, and sociocultural perspectives, this framework provides a more comprehensive account of how prenatal alcohol exposure shapes brain development prior to behavioural recognition. The placenta–brain axis further illustrates a potential biological pathway through which early environmental influences may contribute to later neurodevelopmental outcomes.

Reliance on behavioural presentation may contribute to delayed recognition, misinterpretation, and the accumulation of secondary developmental consequences. At the same time, advances in biological research highlight emerging opportunities for earlier identification and more developmentally informed approaches to support.

The developmental–diagnostic gap provides a clinically relevant and empirically testable framework with implications for research, practice, and public health. Addressing this gap has the potential to improve early identification, reduce secondary harm, and support more effective, developmentally informed intervention across the lifespan.

## References

[ref1] American Psychiatric Association (2013). Diagnostic and Statistical Manual of Mental Disorders. 5th Edn Washington, DC: American Psychiatric Publishing.

[ref2] Bale T. L. (2015). The placenta and neurodevelopment: linking maternal stress to fetal brain development. Neuropsychopharmacology 41, 207–218. doi: 10.1038/npp.2015.231, 26250599 PMC4677129

[ref3] Bariselli S. Lovinger D. M. (2021). Corticostriatal circuit models of cognitive impairments induced by fetal exposure to alcohol. Biol. Psychiatry 90, 516–528. doi: 10.1016/j.biopsych.2021.05.014, 34281711 PMC8463431

[ref4] Center on the Developing Child (2007). InBrief: The Science of Early Childhood Development. Cambridge, MA: Harvard University.

[ref5] Guerri C. Bazinet A. Riley E. P. (2009). Fetal alcohol spectrum disorders and alterations in brain and behaviour. Alcohol Alcohol. 44, 108–114. doi: 10.1093/alcalc/agn105, 19147799 PMC2724862

[ref6] Kolb B. Gibb R. (2011). Brain plasticity and behaviour in the developing brain. J. Can. Acad. Child Adolesc. Psychiatry 20, 265–276, 22114608 PMC3222570

[ref7] Mattson S. N. Bernes G. A. Doyle L. R. (2019). Fetal alcohol spectrum disorders: a review of the neurobehavioral deficits associated with prenatal alcohol exposure. Alcohol Res. Curr. Rev. 40, 1046–1062. doi: 10.35946/arcr.v40.1.01, 30964197 PMC6551289

[ref8] O’Leary C. M. Heuzenroeder L. Elliott E. J. Bower C. (2007). A review of policies on alcohol use during pregnancy in Australia and other English-speaking countries, 2006. Med. J. Aust. 186, 466–471. doi: 10.5694/j.1326-5377.2007.tb00999.x, 17484709

[ref9] Popova S. Lange S. Probst C. Gmel G. Rehm J. (2017). Estimation of the global prevalence of fetal alcohol spectrum disorders. Lancet Glob. Health 5, e290–e299. doi: 10.1016/S2214-109X(17)30021-9, 28089487

[ref10] Riley E. P. Infante M. A. Warren K. R. (2011). Fetal alcohol spectrum disorders: an overview. Neuropsychol. Rev. 21, 73–80. doi: 10.1007/s11065-011-9166-x, 21499711 PMC3779274

[ref11] Rosenfeld C. S. (2021). The placenta–brain axis. J. Neurosci. Res. 99, 271–283. doi: 10.1002/jnr.24603, 32108381 PMC7483131

[ref12] Sessa F. Carotenuto M. Ferorelli D. Esposito M. Pomara C. Salerno M. (2025). Genetic and epigenetic determinants of fetal alcohol spectrum disorders: toward a precision medicine approach. Exp. Mol. Pathol. 144:105002. doi: 10.1016/j.yexmp.2025.105002, 41072308

[ref13] Sessa F. Salerno M. Esposito M. Di Nunno N. Li Rosi G. Roccuzzo S. . (2022). Understanding the relationship between fetal alcohol spectrum disorder (FASD) and criminal justice: a systematic review. Healthcare 10:84. doi: 10.3390/healthcare10010084 35052248 PMC8775242

[ref14] Streissguth A. P. Barr H. M. Kogan J. Bookstein F. L. (2004). Risk factors for adverse life outcomes in fetal alcohol syndrome and fetal alcohol effects. J. Dev. Behav. Pediatr. 25, 228–238. doi: 10.1097/00004703-200408000-00002, 15308923

[ref15] Subramoney S. London L. Fouche J. P. (2018). The early developmental outcomes of prenatal alcohol exposure: a review. Front. Neurol. 9:1108. doi: 10.3389/fneur.2018.01108, 30619064 PMC6305542

[ref16] Wilhelm C. J. Guizzetti M. (2016). Fetal alcohol spectrum disorders: an overview from the glia perspective. Front. Integr. Neurosci. 9:65. doi: 10.3389/fnint.2015.00065, 26793073 PMC4707276

